# Case report: A kidney metastasis from vulvar squamous cell carcinoma

**DOI:** 10.3389/fonc.2023.1280531

**Published:** 2024-01-16

**Authors:** Junwei He, Yuhe Xiao, Lu Wang, Zhaohui Wang, Jun Pan, Zunguang Bai

**Affiliations:** ^1^ The Second Clinical College of Guangzhou University of Chinese Medicine, Guangzhou, China; ^2^ The Second Affiliated Hospital of Guangzhou University of Chinese Medicine, Guangzhou, China; ^3^ Guangzhou University of Chinese Medicine, Guangzhou, China

**Keywords:** vulvar carcinoma, squamous cell carcinoma, renal metastasis, nephrectomy, vulvectomy

## Abstract

**Introduction:**

Distant metastases of vulvar SCC most commonly involve the lung, liver, bone, skin, and lymph nodes. Metastasis from vulvar SCC to the kidneys is extremely rare, with only one case reported in the literature to date.

**Case presentation:**

We report the case of a 53-year-old postmenopausal female patient was diagnosed with vulvar squamous cell carcinoma in an external hospital and following the diagnosis, she had been performed a vulvectomy for squamous cell carcinoma of the vulva, at that time, the patient had not undergone inguinal lymphadenectomy. In July 2019, she was admitted to our hospital due to upper right quadrant pain. An enhanced whole-body CT scan showed a mixed-density tumor of the right kidney with invasion into the right renal portal vein and multiple enlarged retroperitoneal lymph nodes. Positron emission tomography-computed tomography (PET - CT) scan showed a significantly increased radioactivity uptake in the tumor and enlarged lymph nodes, but PET-CT did not show abnormal enlargement of bilateral inguinal lymph nodes and no abnormal increase in radioactivity uptake. PET-CT examination did not show recurrence in terms of local of vulvar. These results led us to be gravely worried about possible renal carcinoma, so it was agreed upon to perform laparoscopic nephrectomy of the right kidney in the same month. Histology of the resected tumor confirmed it to be poorly differentiated squamous cell carcinoma with invasion consistent with metastatic vulvar carcinoma. Based on clinical history, radiological and histological facts, the patient was diagnosed with kidney metastasis from vulvar squamous cell carcinoma. Recovery from surgery went well and the patient was transferred to the oncology department and underwent a chemotherapy regimen consisting of paclitaxel and nedaplatin for further treatment. After 6 courses of chemotherapy. For a year after treatment, the patient had lived progression-free. Unfortunately, she died of tumor progression in July 2022.

**Conclusion:**

Although renal metastasis from vulvar SCC is rare, renal metastasis should be considered for the patient with a history of vulvar cancer, whenever a mass is identified in the kidney. Timely surgical removal of renal metastasis may prolong the survival time.

## Introduction

According to previous studies, vulvar cancer represents 3-5% of malignancies among in the gynecologic genital tract (1). The most common histological type of vulvar cancer is squamous cell carcinoma (SCC). This disease is most commonly diagnosed in postmenopausal women between the ages of 65 to 80, amongst which the occurrence of distant metastases is very rare. The prognosis for vulvar SCC is related to its size, location, degree of cell differentiation, whether lymph node metastasis occurred and the therapeutic measures taken (2). Distant metastases of vulvar SCC most commonly involve the lung, liver, bone, skin, and lymph nodes. Metastasis from vulvar SCC to the kidneys is extremely rare, with only one case reported in the literature to date (3). In this report, we shall discuss a rare case of renal metastasis from a primary vulvar SCC.

## Case presentation

**Figure 1:**
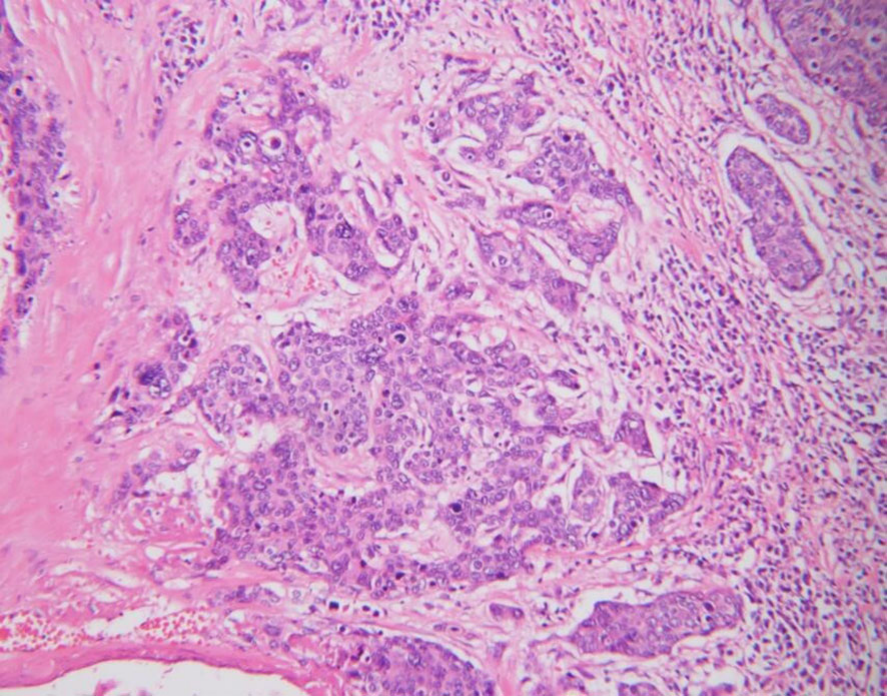
Vulvar tumor showing poorly differentiated squamous carcinoma cell (H&E, 200×).

In 2016, a 53-year-old postmenopausal female patient was diagnosed with vulvar squamous cell carcinoma in an external hospital and following the diagnosis, she had been performed a vulvectomy for squamous cell carcinoma of the vulva, at that time, the patient had not undergone inguinal lymphadenectomy. During postoperative pathological examination, the vulvar tumor has been confirmed to be poorly differentiated squamous cell carcinoma (**Figure 1**), the pathological stage was Ib. The patient also received postoperative adjuvant radiotherapy and chemotherapy, in which the radiotherapy regimen was 50 Gy, while the concurrent chemotherapy drug was cisplatin weekly regimen, 40 mg/m^2^. She remained in remission for 3 years after surgery.

**Figure 2:**
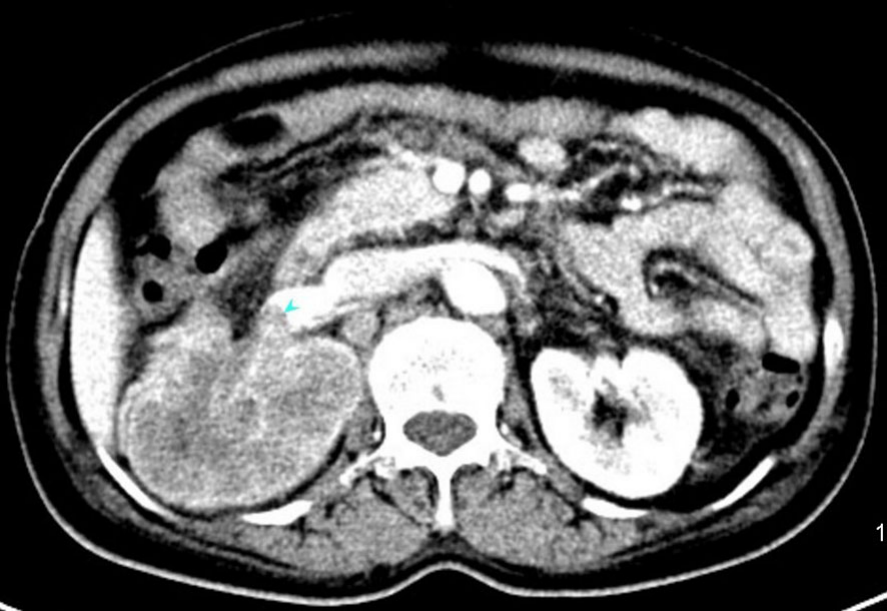
An enhanced computed tomography (CT) scan showing a mixed density tumor of the right kidney measuring 6.6×7.0×9.4cm with invasion into right renal portal vein.

**Figure 3:**
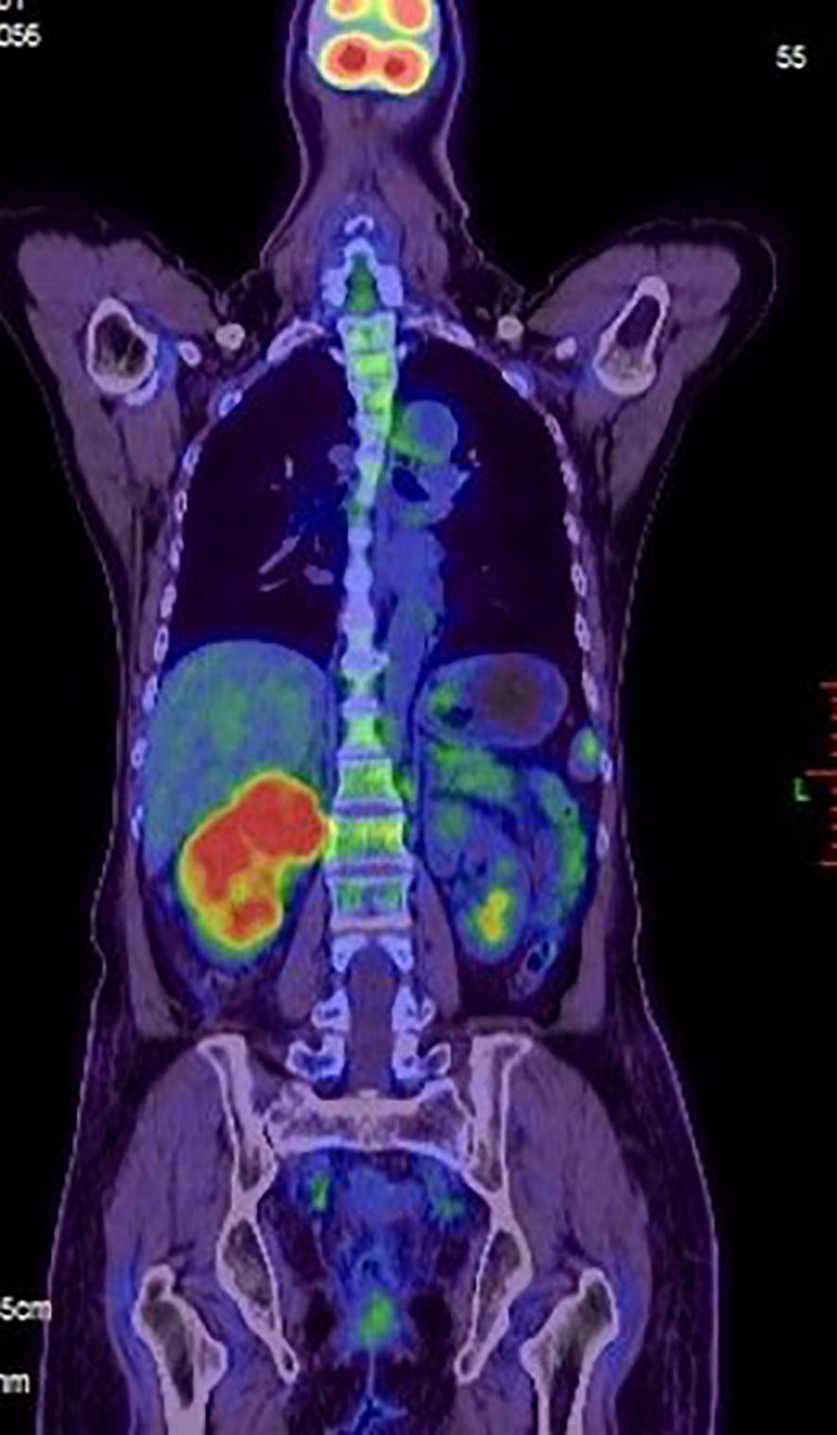
A positron emission tomography-computed tomography (PET-CT) scan was made. Evidence of intense FDG uptake in the tumor of the right kidney.

**Figure 4:**
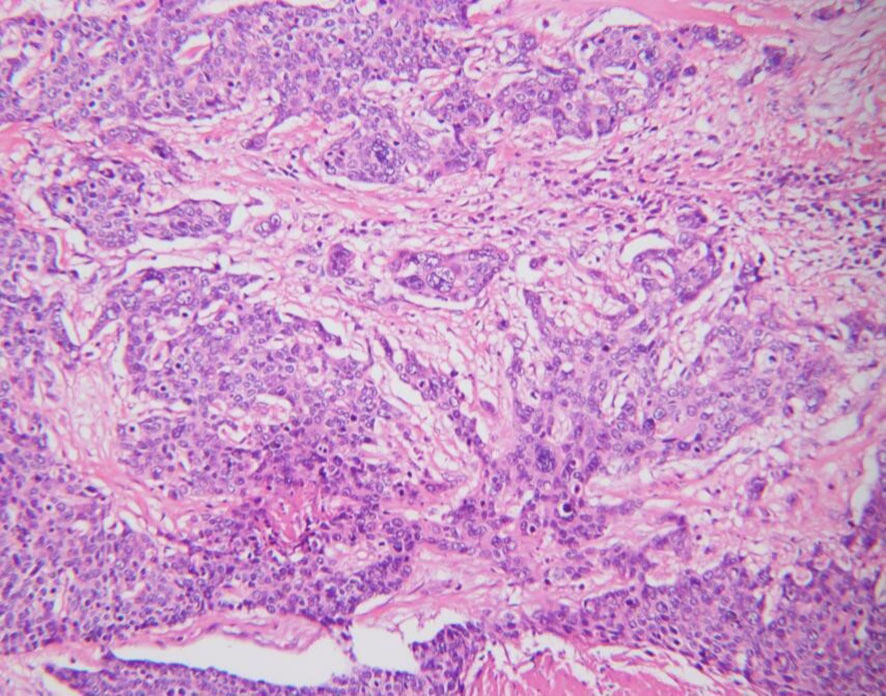
Postoperative histopathology of kidney showing poorly differentiated.

In July 2019, she was admitted to our hospital due to upper right quadrant pain. Physical examination of the patient at that time showed no superficial enlargement of the systemic lymph nodes, especially the inguinal lymph nodes. An enhanced whole-body CT scan showed a mixed-density tumor of the right kidney with invasion into the right renal portal vein and multiple enlarged retroperitoneal lymph nodes (**Figure 2**). Positron emission tomography-computed tomography (PET - CT) scan showed a significantly increased radioactivity uptake in the tumor and enlarged lymph nodes (**Figure 3**), but PET-CT did not show abnormal enlargement of bilateral inguinal lymph nodes and no abnormal increase in radioactivity uptake. PET-CT examination did not show recurrence in terms of local of vulvar. These results led us to be gravely worried about possible renal carcinoma, so it was agreed upon to perform laparoscopic nephrectomy of the right kidney in the same month. Histology of the resected tumor confirmed it to be poorly differentiated squamous cell carcinoma with invasion consistent with metastatic vulvar carcinoma (**Figure 4**). Based on clinical history, radiological and histological facts, the patient was diagnosed with kidney metastasis from vulvar squamous cell carcinoma. Recovery from surgery went well and the patient was transferred to the oncology department and underwent a chemotherapy regimen consisting of paclitaxel and nedaplatin for further treatment. After 6 courses of chemotherapy, CT showed that the enlarged intractraperitoneal lymph nodes had reduced in size. For a year after treatment, the patient had lived progression-free. Unfortunately, she died of tumor progression in July 2022. In the end, the patient lived for 3 years since the discovery of renal metastasis of vulvar SCC.

## Discussion

Vulvar carcinoma represents about 5% of all female genital tract malignancies and the incidence increases with age and peaks at age 70. There are two etiologic pathways for vulvar squamous cell carcinomas namely the human papillomavirus (HPV) - mediated pathway and the HPV - independent pathway, while the HPV-independent pathway accounts for 60% of diagnosed cases (4). A retrospective study of vulvar cancer showed that the 2-year-OS rate of patients who had metastasized after the diagnosis of metastasis was 11.3%, and the median survival period from the first diagnosis of metastasis is 5.6 months (5). Prognosis depends on the stage at age, surgical pathological stage, histological differentiation, as well as the presence of lymph node metastases (6, 7). Another retrospective multicenter study evaluating 502 cases of vulvar cancer indicated that the 5-year survival rate was only 15% for distant recurrences (8). The most frequent distant metastases are pulmonary and hepatic metastases, but renal metastases are seldom. Distant metastases are rare in vulvar carcinoma with an incidence rate of 2-7% as shown in previous studies (8, 9). Due to a lack of prospective trial data, the treatment of vulvar cancer is still controversial, as there is no unified standard regimen. Extensive local excision is considered to be the best treatment for early-stage vulvar cancer, but there is still a risk of local recurrence if surgery is inappropriate (10). Treatment for advanced vulvar cancer includes multimodal therapies which consist of neoadjuvant radiotherapy and chemotherapy and are highly individualized. When radiotherapy or surgery is not feasible, the strategies for treatment usually involve platinum-based systemic combination therapy (11). According the literature, combination chemotherapy is considered more effective than monotherapy. With the use of combination regimens, chemotherapy patients achieved response rates of 40 - 56% (12, 13). With the development of molecular basic research on tumor cell immune recognition and immune regulation, immunotherapy in vulvar cancer has aroused great interest in recent years. Immunotherapy may be a viable alternative to the current treatment for vulvar squamous cell carcinomas (14). Vulvar cancer is characterized by a series of immune evasion mechanisms which are aggressive and lead to a range of symptoms in the organism (15). Due to limited relevant pathological reports, data on the role of immune checkpoint inhibitors in vulvar cancer are often obtained from trials involving other types of tumors. However, treatment with single immune checkpoint inhibitors indicates an overall lack of significant effectiveness based on the data (16, 17).

A study by the University of Texas MD Anderson Cancer Center, which indicated that there were only 151 cases of renal metastasis reported at their facilities from November 1985 to November 2013 (18). Kidney metastasis commonly occurs through a combination of lymphatic and venous routes. Renal metastases are rare, and most studies are only found in the form of case reports through literature search. With only having one reported case in the literature to date, renal metastases from vulvar SCC can be considered extremely rare. In the first case that was reported, the patient had a 24-month recurrence-free survival time after radical surgery and chemo-radiation of vulvar cancer relapse (3). She was treated with radical vulvectomy and chemo-radiation therapy due to vulvar SCC and remained in remission for 24 months. In 2010, she was diagnosed with metastatic squamous cell carcinoma from the vulva to the kidney and underwent nephrectomy. After nephrectomy and paraortic lymph node dissection, the patient’s health declined progressively, and, unfortunately, passed away 2 weeks later.

From current medical standards, treatment patterns for renal metastases are not clear and only appear in individual case reports. Adamy et al. stated in their article, that over the last two decades, 13 patients underwent nephrectomy for metastasis to the kidney, in which 5 cases of the primary tumors were lung cancer. They concluded that nephrectomy can be offered for highly selected patients as a therapeutic option, which positively affected the survival rate of selected patients with renal metastasis (19).

In our report, the patient had a 36-month recurrence-free survival time after the vulvectomy for vulvar SCC, but renal metastasis and retroperitoneal lymph nodes metastasis recurred 3 years later. The primary treatment was not perfect because no inguinal lymph node dissection was performed after the vulvectomy. Panici PB et al. reported nodal status represents a very important prognostic factor in patients of vulvar SCC (20). The recent research suggests that patients with early stage unifocal vulvar SCC(<4 cm) and no suspicious and/or enlarged lymph nodes at imaging should be considered for sentinel lymph node biopsy (21).

We determined the most appropriate treatment plan for the patient after performing a nephrectomy, which comprised a paclitaxel and nedaplatin combination chemotherapy. Following 6 courses of chemotherapy, the CT scan showed that the enlarged lymph nodes had reduced in size. The patient had an one-year progression-free survival. Unfortunately, she died of tumour progression in July 2022. In the end, the patient lived for 3 years since the discovery of renal metastasis of vulvar SCC.

The treatment outcome of a recurrent disease in vulvar SCC is disappointing, and the prognosis of distant metastasis is poor. Since the discovery of renal metastasis of vulvar SCC, the patient has survived for 3 years, which is significantly longer than what has been reported in the literature to date. It is suggested that our surgical resection of the right kidney and combination chemotherapy is still of great significance for these patients and are worthy of clinical application.

## Conclusions

To the best of our knowledge, our report is the second case of vulvar SCC metastasis to the kidney. The diagnosis was based on clinical history, radiological and histological facts. Although renal metastasis from vulvar SCC is rare, renal metastasis should be considered for the patient with a history of vulvar cancer, whenever a mass is identified in the kidney. Timely surgical removal of renal metastasis may prolong the survival time.

## Data availability statement

The original contributions presented in the study are included in the article/supplementary material. Further inquiries can be directed to the corresponding author.

## Ethics statement

The studies involving humans were approved by Ethics Committee of Guangdong Provincial of Chinese Medicine. The studies were conducted in accordance with the local legislation and institutional requirements. Written informed consent for participation in this study was provided by the participants’ legal guardians/next of kin. Written informed consent was obtained from the individual(s) for the publication of any potentially identifiable images or data included in this article.

## Author contributions

JH: Writing – original draft. YX: Writing – original draft. LW: Investigation, Resources. ZW: Supervision, Validation. JP: Formal analysis, Writing – review & editing. ZB: Validation, Project administration.
